# MiR-3194-3p Inhibits Breast Cancer Progression by Targeting Aquaporin1

**DOI:** 10.3389/fonc.2020.01513

**Published:** 2020-08-07

**Authors:** Min Wei, Hailang Yu, Cuixia Cai, Rui Gao, Xuhua Liu, Huimin Zhu

**Affiliations:** ^1^Clinical Laboratory, Nanshan Maternity and Child Healthcare Hospital, Shenzhen, China; ^2^Department of Science and Education, Nanshan Maternity and Child Healthcare Hospital, Shenzhen, China; ^3^Department of Biochemistry and Molecular Biology, School of Basic Medical Sciences, Southern Medical University, Guangzhou, China

**Keywords:** miR-3194-3p, AQP1, proliferation, migration, invasion, apoptosis, breast cancer

## Abstract

Increasing evidence indicates that the Aquaporin1 (AQP1) aberrant expression may be related to a wide variety of human cancers, including breast cancer (BC). In the present study, we explore the effects and possible mechanism of miR-3194-3p on the biological behaviors of BC. At first, miR-3194-3p is found to modulate *AQP1* expression targeting the 3′-UTR using miRNA target prediction algorithms. MiR-3194-3p expression is markedly downregulated, and *AQP1* expression is upregulated in BC tissues compared with adjacent normal breast tissues. Moreover, the differential expression of miR-3194-3p and *AQP1* are observed in four BC cells with different malignancy degree. Meanwhile, a significant negative correlation between *AQP1* and miR-3194-3p expressions in tumor tissues from 30 BC patients is revealed. miR-3194-3p mimic remarkably inhibits cell proliferation, migration, and invasion as well as promotes apoptosis in MDA-MB-231 cells while miR-3194-3p inhibitors exert an opposite role in MCF-7 cells. Dual-luciferase reporter system demonstrates that *AQP1* is a direct target gene of miR-3194-3p. Overexpression of *AQP1* by pBABE-puro-*AQP1* vector partially abrogates the effect of miR-3194-3p mimic in MDA-MB-231 cells. In short, our results suggest that miR-3194-3p suppresses BC cell proliferation, migration, and invasion by targeting *AQP1*, providing a novel insight into BC tumorigenesis and treatment.

## Introduction

The incidence and mortality of breast cancer (BC) have been high among female malignant tumors, which seriously threatens the health of women worldwide (1, 2). Despite advances in early screening and therapeutic strategy for BC in recent years, the 5-year survival rate of patients with BC is still not ideal due to high metastases and recurrences (3). Therefore, a deep understanding of the molecular mechanisms of BC is essential to develop new treatments and improve prognosis for BC.

Aquaporin1 (AQP1) is a kind of membrane channel protein widely existing in endothelial and epithelial cells of human tissues, which can efficiently and specifically transport water molecules (4). A growing number of studies have found that AQP1’s function is not limited to transporting water molecules; it is closely related to a wide variety of tumors, including tumors of brain, prostate, breast, ovary, colon, and lung (5). It has been reported that AQP1 is involved in cell migration (6), angiogenesis (7), and tumor growth (8). Especially in BC, AQP1 overexpression is significantly associated with poor prognosis in aggressive breast cancer (9). Furthermore, Esteva-Font et al. found that AQP1 deficiency reduces vessel density and lowers lung metastases in mice models (10). However, the possible regulation mechanisms involved in AQP1 overexpression in BC are still unclear.

MicroRNA (miRNA) is a class of endogenous non-coding RNAs with a length of 19–25 nucleotides that negatively regulate target gene expression at the post-transcriptional level (RNA cleavage or translation repression) mainly by binding to the complementary region on the 3′-untranslated regions (3′-UTR) of target gene mRNA (11, 12). miRNAs plays an important role in cell proliferation, differentiation, and apoptosis, and its abnormal expression may be closely related to tumorigenesis (13). Emerging research continues to focus on miRNAs as potential markers for BC targeted therapy and patient prognosis prediction (14). Recent studies reveal that miRNAs may act as oncogenes or suppressor genes in BC by participating in different cellular pathways (15, 16), including via targeting *AQP1*. Lately, miR-3194-3p was found significantly downregulated in hepatocellular carcinoma (HCC) tissues and cell lines, which was associated with metastasis and recurrence of HCC through targeting BCL9 (17). Nevertheless, the function of miR-3194-3p and its relationship with *AQP1* in BC remain unknown.

To obtain a comprehensive understanding of the role and possible mechanism of miR-3194-3p in BC progression, we explored the effects of miR-3194-3p upregulation and downregulation on cellular biological process of BC and identified *AQP1* as a direct target of miR-3194-3p. Moreover, the alteration of miR-3194-3p expression and its effect on BC cell growth were observed by promoting *AQP1* expression. The expression pattern and biological role of miR-3194-3p and its target gene *AQP1* in BC have not yet been elucidated, and our findings suggest that miR-3194-3p is a novel and promising molecular target for BC treatment.

## Materials and Methods

### Clinical Samples

BC tumor tissue and paired adjacent tissue were obtained from 30 patients with BC at the Nanfang Hospital of Southern Medical University (Guangzhou, China). Tissue specimens were collected from female patients with primary BC who underwent surgical resection between September 2014 and December 2016. The BC tissues removed surgically were placed in RNAlater (Ambion, Austin, TX, United States) immediately at 4°C for at least 24 h and stored at −80°C until used. This study was conducted in accordance with the Helsinki Declaration and was approved by the Ethics Committee of Nanfang Hospital of Southern Medical University.

### Cell Culture and Transfection

Four BC cell lines (MDA-MB-231, MCF-7, ZR-75-1, SKBR3) and a normal human mammary epithelial cell line (MCF-10A) were obtained from the Chinese Academy of Sciences (Shanghai, China). All cell lines were cultured in DMEM medium (Thermo Fisher Scientific, United States) supplemented with 10% fetal bovine serum (Gibco, United States) and 1% penicillin, and incubated at 37°C and 5% CO_2_. The oligonucleotides of miR-3194-3p mimic (miR-3194-3p) and its negative control (mi-NC), miR-3194-3p inhibitor (anti-3194-3p) and its negative control (anti-NC) were purchased from GenePharma (Shanghai, China). A pBABE-puro-*AQP1* expression vector was constructed and stored in the department of biochemistry and molecular Biology of Southern Medical University (18). For transfection, cells were seeded onto plates and incubated for 15 h and then transfected using Lipofectamine 2000 reagent (Invitrogen, Carlsbad, CA, United States) following the manufacturer’s instructions.

### RNA Isolation, RNA-Reverse Transcription, and qRT-PCR Analysis

Expression levels of miR-3194-3p and *AQP1* mRNA in BC tissues and BC cells were detected by qRT-PCR analysis following the protocols of previous studies (15). Total RNA was isolated using TRIzol reagent (LifeTechnologies, Carlsbad, CA, United States). To quantify miR-3194-3p expression, reverse transcription was performed with the Bestar^TM^ qPCR RT Kit (DBI Bioscience, Inc., Germany). miRNA quantification was measured using the SYBR Green qPCR MasterMix (DBI, Bioscience Inc., Germany) on the ABI 7500 qPCR system (Applied Biosystems, Carlsbad, CA, United States) with U6 small nuclear RNA as an internal control. To quantify *AQP1* mRNA expresssion, M-MLV reverse transcriptase (Clontech, Palo Alto, CA, United States) and SYBR Premix Ex Taq GC kit (DBI, Bioscience Inc., Germany) were applied. All primers were designed and synthesized by Invitrogen (Carlsbad, CA, United States). The relative expression of *AQP1* was normalized to glyceraldehyde 3-phosphate dehydrogenase (GAPDH). The relative expression levels of miR-3194-3p and *AQP1* mRNA were calculated using the 2^–ΔΔ*Ct*^ approach.

### Western Blot Analysis

Total protein from BC cells (1 × 10^6^) was extracted with radio immunoprecipitation assay (RIPA) lysis buffer (Beyotime, China). Protein concentration was determined using the Bradford protein assay (Bio-Rad, United States). Take 40 μg/well of protein for 12.5% sodium dodecyl sulfate-polyacrylamide gel electrophoresis (SDS-PAGE), transfer the separated protein to polyvinylidenedifluoride (PVDF) membrane, and block the reaction with blocking solution containing 5% skimmed milk powder at room temperature for 2 h. Anti-AQP1 (1:2000; AF5231; Affinity) and anti-GAPDH (1:1000; ab8245; Abcam) antibodies were added and incubated at 4°C overnight. After washing the PVDF membrane three times with TBST, goat anti-rabbit IgG secondary antibody labeled with horseradish peroxidase (HRP) (1:20,000; BA1054; Boster) was added for 1 h. Protein bands were detected using an enhanced chemiluminescence (ECL) detection system (Amersham, United Kingdom). Image-Pro Plus 6.0 (Media Cybernetics, United States) was used to quantify protein bands, and GAPDH was used as a control.

### Cell Counting Kit-8 Assay (CCK-8)

Cell proliferation was examined using a CCK-8 kit (Dojindo, Japan) according to the manufacturer’s protocol. Cells from each group were harvested at 24 h post-transfection and inoculated into 96-well plates (5 × 10^3^ cells/well). The plates were placed in a cell culture incubator; CCK-8 (10 μL/well) was added after 0, 24, 48, and 72 h, respectively, and then cultured for another 2 h. The absorbance (A) value of each well at 450 nm was measured by microplate spectrophotometer (Bio-Rad, Hercules, CA, United States). Cell growth curves were plotted with time (T) as the horizontal axis and light absorption value (A) as the vertical coordinate.

### 5-Ethynyl-2′-Deoxyuridine (EdU) Assay

Cell proliferation ability was also measured by an EdU assay kit (Ribobio, China) as described previously (19). In 96-well plates, the post-transfected cells, in turn, were incubated with EdU (50 μM) for 2 h, 0.5% Triton X for 20 min, ApolloR reaction cocktail (100 μL) for 30 min, and Hoechst 33342 (100 μL) for 30 min. The cell images were taken using a fluorescence microscope (Olympus Corporation of Japan) at 100× magnification.

### Cell Migration and Invasion Assays

The wound-healing assay was conducted to evaluate cell migration ability: 3 × 10^5^ cells per well were seeded in triplicate into six-well plates and cultured for 24 h. The cell surface was scratched with a pipette tip. The suspended cells were washed off with PBS and cultured with fresh DMEM medium without serum for an additional 24 h. The area of the cell crawling from the scratch toward the center is observed under a microscope, and the size of the area indicates the cell’s ability to migrate (20). The migration distance was analyzed by Image-Pro Plus 6.0 software.

Cell invasion assays were performed using Transwell chambers (8-μm pore size; Corning, United States) coated with Matrigel matrix (BD Biosciences, United States). A detailed experiment was performed similar to that previously reported (21). The invaded cells were counted with a fluorescence inversion microscope (Olympus, Japan) at 200× magnification. Six fields per well were randomly selected under the microscope, and the average number of cells in each field was calculated.

### Flow Cytometry Analysis of Cell Apoptosis

Cell apoptosis was evaluated using the Annexin V-FITC/PI Apoptosis Detection Kit (Kaiji, China) and flow cytometry (Becton Dickinson, United States). After 24 h transfection, cells were collected by digestion with 0.25% trypsin (without EDTA), rinsed, and resuspended in 500 μL binding buffer. Then, 5 μL Annexin V-FITC and 5 μL propidium iodide (PI) were added to the stain. After 10 min light-avoidance reaction, the apoptosis rate of the cells was interpreted by FlowJo software, version 10.0 (Flow Jo, United States).

### Bioinformatic Analysis and Luciferase Reporter Assay

The online miRNA databases MiRanda^1^, miRBase^2^, and TargetScan^3^ were used to search for miRNAs that regulate *AQP1* expression. The wild-type (WT) and mutant-type (MUT) *AQP1* 3′-UTR oligonucleotides miR-3194-3p were cloned into the psiCHECK^TM^-2 vector (Promega, United States). These constructed vectors were cotransfected with miR-3194-3p mimic or mi-NC into HEK293T cells using Lipofectamine 2000. Then, 24 h after transfection, the luciferase activity was detected using a dual-luciferase reporter assay system (Promega, United States).

### Statistical Analysis

All experiments were executed at least three independent times. The statistical data are presented as mean ± standard deviation and analyzed using GraphPad Prism 6.0 (CA, United States). Two-tailed Student’s *t*-tests were conducted to interpret the differences. The correlation between miR-3194-3p and *AQP1* mRNA expressions in BC tissues was assessed with Spearman’s correlation test. *P* value <0.05 was considered statistically significant.

## Results

### MiR-3194-3p and *AQP1* Expression in BC Tissues and Cell Lines

**FIGURE 1:**
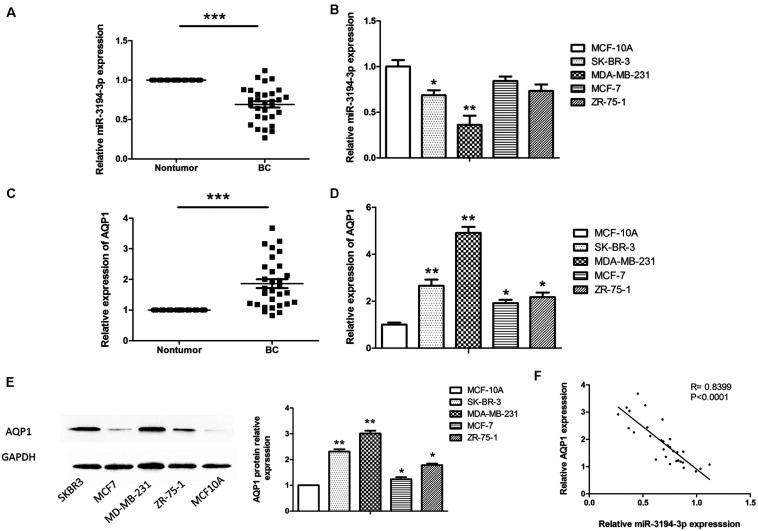
The miR-3194-3p and AQP1 expression levels in BC tissues and cell lines. Comparing differences in the expression levels of miR-3194-3p between **(A)** BC and adjacent non-tumor tissues (*n* = 30); **(B)** BC cell lines and the control breast epithelial cells (MCF-10A); differences in *AQP1* mRNA level between **(C)** BC and adjacent non-tumor tissue; **(D)** BC cell lines and MCF-10A; differences in AQP1 protein level between **(E)** BC cell lines and MCF-10A. **(F)** A significant inverse correlation between miR-3194-3p and AQP1 expression was observed in BC tissues. All PCR reactions were performed independently in triplicate (*P < 0.05, **P < 0.01, and ***P < 0.001).

To investigate the potential role of miR-3194-3p in BC development, the expression levels of miR-3194-3p in BC tissue samples (**n** = 30) and cell lines (MDA-MB-231, MCF-7, ZR-75-1, SKBR3) were measured by qRT-PCR. The results show that the expression of miR-3194-3p is markedly downregulated in BC tissues as compared with that of adjacent non-tumor tissues (Figure 1A). Moreover, we found that miR-3194-3p expression is also significantly lowered in high invasive BC cell lines (MDA-MB-231 and SK-BR-3) compared with a normal human mammary epithelial cell (MCF-10A) (Figure 1B). Conversely, the mRNA and protein of AQP1 in BC tissues and cell lines are significantly higher than those in the adjacent non-tumor tissues (Figure 1C) and MCF-10A cells (Figures 1D,E). It is worth noting that miR-3194-3p expression was lowest and **AQP1** mRNA and protein expression was highest in MDA-MB-231 cells. Spearman’s correlation analysis found a significant negative correlation between the expression levels of miR-3194-3p and **AQP1** mRNA in BC tissues (Figure 1F). These results suggest that miR-3194-3p and **AQP1** expressions are closely involved in BC progression.

### MiR-3194-3 Overexpression Inhibits Proliferation, Migration, and Invasion and Promotes Apoptosis in BC Cells

**FIGURE 2:**
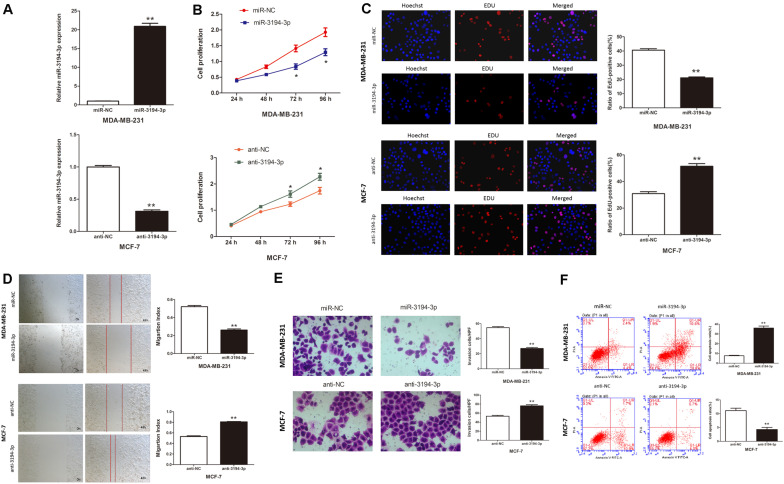
miR-3194-3p expression regulates cell proliferation, migration, invasion, and apoptosis in BC. **(A)** miR-3194-3p expression was measured by qRT-PCR in MDA-MB-231 and MCF-7 cells transfected with miR-3194-3p mimic/inhibitor and their negative controls. Cell proliferation was assessed with **(B)** CCK-8 assay and **(C)** Edu assay. **(D)** Cell migration was measured by wound-healing assay. **(E)** Cell invasion was measured by transwell assay. **(F)** Cell apoptosis was measured by Flow cytometry analysis (*P < 0.05 and **P < 0.01).

To better understand the role of miR-3194-3p on the biological behaviors of BC cells, we subsequently transfected MDA-MB-231 cells that had the lowest miR-3194-3p expression with mimics and MCF-7 cells that had the highest miR-3194-3p expression with inhibitors and their corresponding miRNA controls. qRT-PCR assay revealed miR-3194-3p expression was effectively upregulated in MDA-MB-231 cells and downregulated in MCF-7 cells at 48 h post-transfection (Figure 2A).

The effect of miR-3194-3p on BC cell proliferation was assessed by CCK-8 and EdU assay. The CCK-8 assay indicated that miR-3194-3p overexpression significantly reduced MDA-MB-231 cell viability (Figure 2B). The EdU assay displayed remarkably fewer EdU-positive cells in cells overexpressing miR-3194-3p than in control cells (Figure 2C). The wound-healing assay suggests that the migratory capacity of MDA-MB-231 cells transfected with miR-3194-3p mimics was much weaker than that of the miR-NC group cells (Figure 2D). Similarly, the transwell assay reveals that miR-3194-3p upregulation significantly undermined the invasion potential of MDA-MB-231 cells (Figure 2D). Flow cytometry analysis reveals that miR-3194-3p upregulation promotes apoptosis in MDA-MB-231 cells (Figure 2E). Conversely, miR-3194-3p downregulation promotes cell proliferation, migration, and invasion and inhibits apoptosis in MCF-7 cells transfected with miR-3194-3p inhibitors (Figures 2B–F). Taken together, these data indicate that miR-3194-3p overexpression inhibits BC cell proliferation, migration, and invasion and promotes cell apoptosis.

### MiR-3194-3p Directly Targets and Inhibits *AQP1* Expression

**FIGURE 3:**
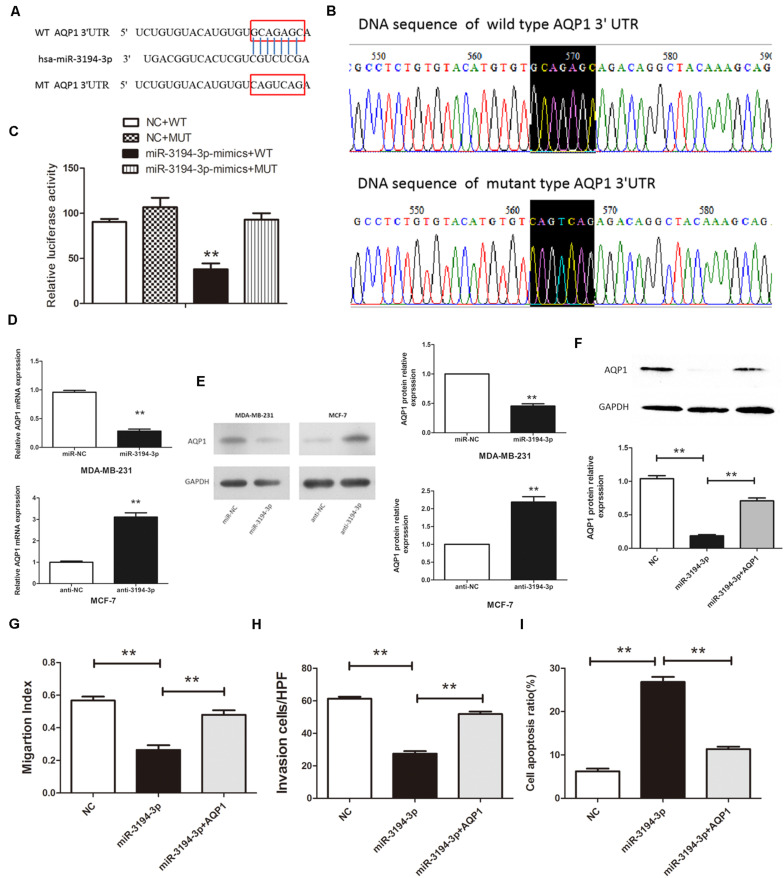
miR-3194-3p negatively regulates the expresssion of *AQP1* by binding to the 3′-UTR of its mRNA. **(A)** The predicted miR-3194-3p-binding site in the *AQP1* 3′-UTR. **(B)** Mutations were generated and sequenced at the putative miR-3194-3p-binding site located in the *AQP1* 3′-UTR. **(C)** Either the WT or MUT reporter plasmid was cotransfected with either miR-3194-3p or NC oligo into HEK293T cells, and the luciferase activity was determined. **(D)** qRT-PCR analysis and **(E)** Western blot analysis of AQP1 expression in MDA-MB-231 and MCF-7 cells transfected with miR-3194-3p mimic/inhibitor and their negative controls. **(F)** Western blot analysis of AQP1 expression in MDA-MB-231 cells cotransfected with pBABE-puro-*AQP1* and miR-3194-3p mimic. **(G)** Cell migration, **(H)** cell invasion, and **(I)** cell apoptosis was measured in MDA-MB-231 cells cotransfected with pBABE-puro-*AQP1* and miR-3194-3p mimic (**P < 0.01).

Our previous study shows that AQP1 has different roles in human cancers, including chronic myeloid leukemia (CML) (18), hepatocellular carcinoma (22), and cervical cancer (23). Another study finds AQP1 is overexpressed in BC tissues and participates in the pathogenesis of BC (24). To further clarify the regulatory mechanism of high AQP1 expression in BC tumorigenesis, we used three bioinformatic algorithms (MiRanda, miRBase, and TargetScan) to search for potential miRNAs that regulate *AQP1* expression. As shown in Figure 3A, miR-3194-3p can directly bind with the *AQP1* 3′-UTR. The MiR-3194-3p target region within *AQP1* 3′-UTR ranges from 30,924,372 to 30,924,393 bp in the genomic sequence of *AQP1* (NC_000007.14, location: 30,911,694–30,925,517).

To confirm that *AQP1* is a direct target of miR-3194-3p, WT and MUT *AQP1* 3′-UTR transcription reporter vectors were constructed and transfected into HEK293T cells (Figure 3B). As pointed out in Figure 3C, high expression of miR-3194-3p significantly inhibits the luciferase activity of WT-*AQP1*-3′-UTR, whereas the luciferase activity of MUT-*AQP1*-3′-UTR is unaffected. Meanwhile, qRT-PCR and Western blot analyses prove that both mRNA and protein levels of AQP1 are significantly lowered in miR-3194-3p overexpressing MDA-MB-231 cells and higher in miR-3194-3p knockdown MCF-7 cells (Figures 3D,E). Altogether, *AQP1* is the direct target gene of miR-3194-3p, and miR-3194-3p affects the progression of BC cells by downregulating *AQP1* expression.

### *AQP1* Overexpression Partly Abrogates the Effect of MiR-3194-3p Mimic

To further elucidate that miR-3194-3p affects BC cell migration and invasion capacity is mediated by *AQP1*, we restored *AQP1* in miR-3194-3p overexpressing MDA-MB-231 cells. Cells were transfected with pBABE-puro-*AQP1* retroviral vector and miR-3194-3p mimic at the same time. As shown in Figure 3F, the suppression effect of miR-3194-3p mimic on *AQP1* gene expression was reversed by pBABE-puro-*AQP1*. As expected, the effect of the miR-3194-3p mimic on cell migration, invasion, and apoptosis was significantly reversed by *AQP1* overexpression (Figures 3G–I). Our findings support that miR-3194-3p regulates BC cell migration, invasion, and apoptosis through the *AQP1* signaling pathway.

## Discussion

Accumulating research has found that AQP1 can affect multiple important biological processes, including angiogenesis, wound healing, tumor metastasis, and invasion, etc. (25). AQP1-mediated rapid transmembrane transport of water flux is one of the important mechanisms to promote tumor cell migration (26). Concrete evidence shows that, after subcutaneous or intracranial tumor cell implantation, tumor growth is significantly inhibited in *AQP1*-deficient mice with reduced tumor vasculature and extensive necrosis (27). By establishing a mouse melanoma model, specific *AQP1* siRNAs can effectively reduce the density of tumor microvessels, showing less obvious local tumor invasion and tumor metastasis in *in vivo* experiments (28). Otterbach et al. reports that high expression of AQP1 in BC is significantly associated with basal-like phenotype and poor survival (9). And in a long-term follow-up, *AQP1* expression in 203 patients with aggressive BC shows a significant correlation with high tumor grade, medullary-like histology, and “triple-negativity” (29). Although evidence suggests that *AQP1* may be a potentially promising target for inhibiting metastasis and aggressiveness in BC, the regulatory mechanisms of its aberrant expression have not been fully elucidated (7).

So far, approximately 50% of annotated miRNAs in humans are located in cancer-associated fragile loci and other genomic regions, and those miRNAs may act as both oncogenes or anti-oncogenes. Furthermore, it is estimated that more than 5,000 human genes, 30% of which have been validated, can serve as target genes of those miRNAs (30) and are mainly involved in the cellular signaling pathways regulating diverse biological processes, including metabolism (31), apoptosis (32), and the cell cycle (33).

To date, some miRNAs targeting *AQP1* have been verified in a variety of tumors. For instance, miR320 is downregulated in plasma and tumor tissues of BC patients, and its target gene *AQP1* is highly expressed, both of which are associated with poor prognosis (24). Overexpression of miR-495 may activate the p38 MAPK signaling pathway, inhibit *AQP1*, and promote the proliferation and differentiation of osteoblasts in tibial fracture mice (34). In thyroid cancer, it is reported that miR-223 inhibits cell proliferation and induces apoptosis by regulating *AQP1* expression (35).

Here, we find miR-3194-3p regulates cell proliferation, migration, and apoptosis in BC by targeting *AQP1*. First, we predicted that miR-3194-3p may have a specific regulation on *AQP1* through bioinformatics methods, which next was verified by qRT-PCR and luciferase reporter assay. We also searched gene databases, such as NCBI and PolymiRTS, and found no gene polymorphism in the region of miR-3194-3p-*AQP1* interaction. The presence/absence of these polymorphisms in the miRNA seed region or target gene region may influence the loss/creation of miRNA binding sites, thus affecting the regulatory role of miRNA. The genetic variants of *AQP1* 3′-UTR and their effects on miR-3194-3p regulation remain to be further discovered.

In our study, miR-3194-3p expressions are remarkably lower in BC tissues, and the expression of *AQP1* mRNA and protein are significantly higher in BC tissues compared with adjacent normal tissues. There is a significant negative correlation between miR-3194-3p and *AQP1* expression in 30 cases of breast cancer and adjacent tissues (*r* = −0.84, *P* < 0.01). As is well known, BC with different molecular subtypes has significant differences in treatment effects and prognosis. Therefore, we compared the expression of miR-3194-3p and *AQP1* in multiple BC cell lines. The results show that miR-3194-3p expression is lowest and *AQP1* expression is highest in triple-negative MDA-MB-231 cells. All these findings suggest that miR-3194-3p and *AQP1* are involved in carcinogenesis and may be associated with the tumor malignancy of BC. The relationship between miR-3194-3p and clinicopathological characteristics and prognosis of BC remains to be further investigated.

To further investigate whether miR-3194-3p suppresses BC progression via regulating *AQP1*, MDA-MB-231 and MCF-7 cells were transfected with miR-3194-3p mimic or inhibitors, and the ability of BC cells to grow, migrate, and invade and apoptosis were measured. Gain- and loss-of-function experiments reveal that miR-3194-3p overexpression prominently inhibits the proliferation, migration, and invasion of BC cells, and miR-3194-3p knockdown increases these cellular biological behaviors *in vitro*. Moreover, the cell apoptotic rate was notably increased by miR-3194-3p mimic. Importantly, we subsequently applied the *AQP1* overexpressed vector in MDA-MB-231 cells and found that *AQP1* upregulation partly abrogated the effect of miR-3194-3p mimic on cell migratory ability. These results strongly suggest that miR-3194-3p serves as a novel tumor suppressor through regulating *AQP1*. However, to fully understand the regulatory mechanism of miR-3194-3p in the pathogenesis of BC, further research is needed.

## Data Availability Statement

The raw data supporting the conclusions of this article will be made available by the authors, without undue reservation.

## Ethics Statement

The studies involving human participants were reviewed and approved by the Ethics Committee of NanFang Hospital of Southern Medical University. The patients/participants provided their written informed consent to participate in this study.

## Author Contributions

MW, HY, and CC took part in research design, conducted experiments, and drafted the manuscript. RG, XL, and HZ participated in the data analysis and helped to draft the manuscript. All authors read and approved the final manuscript.

## Conflict of Interest

The authors declare that the research was conducted in the absence of any commercial or financial relationships that could be construed as a potential conflict of interest.

## Footnotes

1 http://www.microrna.org/microrna/home.do

2 http://www.mirbase.org/

3 http://www.targetscan.org
